# Prevalence of Temporomandibular Disorder-Related Pain among Adults Seeking Dental Care: A Cross-Sectional Study

**DOI:** 10.1155/2022/3186069

**Published:** 2022-09-05

**Authors:** Qoot Alkhubaizi, Mai E. Khalaf, Afnan Faridoun

**Affiliations:** Department of General Dental Practice, Faculty of Dentistry, Health Sciences Center, Kuwait University, Kuwait City, Kuwait

## Abstract

**Objectives:**

Temporomandibular disorders (TMD) are a constellation of painful conditions that affect the craniofacial complex. The etiology and risk factors of these conditions have been extensively studied; however, the data available describing the epidemiology of TMD in the Middle East are scarce. We aimed to estimate the prevalence and risk factors of TMD-related pain in a sample population of dental school clinic patients.

**Methods:**

This observational cross-sectional study used a translated and culturally adapted temporomandibular disorders pain screener, a part of the Diagnostic Criteria/Temporomandibular Disorders (DC/TMD) criteria instrument, and questions related to demographic characteristics and risk factors for TMD. Data were analyzed through chi-square and Mann–Whitney *U* tests using SPSS.

**Results:**

The sample population included 199 participants (66% female and 34% male). The prevalence of TMD-related pain was 26.8% (*n* = 42); men and women did not differ statistically in their TMD-related pain. TMD-related pain cases differed significantly on the Center for Epidemiologic Studies Depression Scale (CES-D) and body pain scores compared with noncases.

**Conclusion:**

The prevalence of TMD-related pain in the clinical sample population was high. Hence, the onus is on dental health services to screen and educate patients on TMD conditions regularly.

## 1. Introduction

Temporomandibular joint (TMJ) disorders (TMD) collectively describe a wide array of acute and chronic pain conditions affecting the craniofacial and oral complexes [1]. Signs and symptoms of these disorders range from tenderness in the muscles of mastication to severe TMJ disability, which can affect both hard and soft tissue components of the TMJ and include limited mandibular range of motion, joint pain, limited mouth opening, and mandibular deviation during function [1]. Despite the inconsistencies in estimating the current burden of TMD, population surveys investigating painful conditions affecting the head and neck area in European and North American societies have reported that TMD was ranked second to headache. For every 100,000,000 full-time working adults in the United States (US), TMD accounts for the loss of 17,800,000 workdays [2]. Furthermore, it poses a financial strain of approximately $4 billion annually in the USA [3]. Estimates of the prevalence of TMD among adults vary by geography, setting, and age range. In Saudi Arabia, the prevalence of TMD among university students, with ages ranging between 20 and 25, was 37%, while in Lebanon estimates were between 19.7 and 59.5% based on urban versus clinical sampling settings [4, 5]. In Sweden, the relative risk (RR) of TMD among patients in a chronic pain clinic was RR = 7.1 (95% CI 5.9–8.4) [6]. While in Poland, the prevalence of TMD in an urban setting was reported to be 55.9% [7]. A systematic review and meta-analysis that included 21 studies investigating examiner verified TMD prevalence reported estimates from 4.6% to 31% for adults [8]. The variety of published data is a testament to the lack of agreement in the scientific community.

A thorough understanding of the multifactorial etiologies associated with TMD is crucial for the diagnosis and delivery of judicious treatment [1]. Although some risk factors for TMD have been investigated and determined in the “Orofacial Pain: Prospective Evaluation and Risk Assessment” (OPPERA) project in the USA, the etiology of TMD is poorly understood [9]. This has been attributed mainly to differences in research methodologies, study samples, and clinical definitions of the condition. However, there is consensus that TMD is often associated with several risk factors, which include sex; there seems to be a significant female predilection. Although debated, the condition is reported to be 2–3 times more likely to develop in women than in men [1, 9–14]. Moreover, individuals with depressive symptoms complain more frequently of TMD pain [7, 15–17]. While TMD is viewed within the context of the psychosocial mode, the relationship between occlusion and development of TMD as a causal factor is controversial. A recent systematic review revealed that there was no evidence for occlusal disharmony as a causing factor of TMD [13, 18–23]. TMD pain and symptoms are also more prevalent in patients with rheumatological conditions, such as systemic sclerosis [24].

Screening instruments, such as surveys and questionnaires, are identification tools for patients warranting referral for further investigation and intervention [16]. A number of TMD screening instruments are available in the literature; however, none have been translated and culturally adapted to the Arabic language, with the exception of Research Diagnostic Criteria for Temporomandibular Disorders (RDC/TMD) [25]. The latter is a comprehensive instrument that covers a myriad of manifesting signs and symptoms of TMD and is useful in both research and clinical settings. However, the instrument is lengthy; it can be expensive and time-consuming in a primary care setting. Revisions to Axis I of the RDC/TMD led to the development of the Diagnostic Criteria/Temporomandibular Disorders (DC/TMD), which is more user-friendly [14]. The temporomandibular disorders pain screener (TMD-pain screener) at its inception was an independent instrument, which was later incorporated within the overall DC/TMD protocol. It is a shorter instrument with good validity compared to the RDC/TMD protocol. Validation studies reported a 99% sensitivity and 97% specificity of the TMD-pain screener in detecting the presence or absence of TMD-related pain [16].

Regionally, a few cross-sectional studies have described the prevalence of signs and symptoms of TMD using validated and culturally adapted instruments. Controlling risk factors is crucial in the management of TMD, highlighting the need to establish disease burden estimates. It is also imperative that the community-level risk factors are recognized for each region. This study aimed to estimate the prevalence of TMD-related pain in patients visiting the Kuwait University Dental Center (KUDC) and to determine the characteristics of patients who reported TMD-related pain in the sample population.

## 2. Materials and Methods

This cross-sectional study was conducted using a convenience sampling process for adult patients visiting the admission department of the Kuwait University Dental Center (KUDC). The KUDC clinic provides free dental care to the entire Kuwaiti population, regardless of their nationality and location of residence. The study was approved by the Health Sciences Center (HSC) Ethical Committee of Kuwait University, in full accordance with the World Medical Association Declaration of Helsinki, approval number: VDR/EC/2269. Informed consent was obtained from all participants involved in the study.

In total, 199 participants were enrolled in this study. The inclusion criteria were as follows: (1) adults aged 18+ years, (2) ability to comprehend Arabic and/or English language, (3) fully dentate, and (4) males and females. The exclusion criteria were as follows: (1) acute odontogenic pain, (2) history of orthodontic treatment, (3) history of facial trauma, (4) treatment with the occlusal guard, and (5) pregnancy (self-reported). Data were collected over a period of six months, from February 2019 to August 2019.

The questionnaire included 37 questions investigating demographics such as age, sex, education level, governorates of residence, TMD-pain screener, pain in other joints, and the Center for Epidemiologic Studies Depression Scale (CES-D).

### 2.1. Questionnaire Variables

#### 2.1.1. Temporomandibular Disorders Pain Screener (TMD-Pain Screener)

The dependent variable was TMD-related pain as measured by the short version [16]. The screener was translated and culturally adapted to Arabic using the standard protocol outlined by the Committee for Translations and Protocols of the International Network for Orofacial Pain and Related Disorders Methodology (INfORM). The screener is part of the Diagnostic Criteria for Temporomandibular Disorders (DC/TMD), an extensive diagnostic tool based on a standardized assessment that can be used in both clinical and research settings. DC/TMD has been extensively translated into different languages using a protocol with accepted standards [14, 26].

The screener is composed of three items inquiring about the presence or absence of pain and stiffness in the jaw and temple areas within the last 30 days. In addition, it is used to investigate the effects of daily activities that necessitate the use of the TMJ, such as chewing, kissing, and talking. Items 1–3A represent the short screener, and items 1–3D represent the long screener. Responses “a,” “b,” and “c” correspond to 0, 1, and 2 points, respectively. The possible scores for this scale range from 0 to 7. The threshold values for the short version were a score of 2 and above, and for the long version, the values were 3 and above.

### 2.2. Pain in Other Joints

Participants were asked six questions about neck, knee (right and left), hip (right and left), and back pain. We used the participants' responses to these six questions on bodily pain to determine the extent of pain that could reasonably be associated with joint-related pathology. The potential answers to the questions were yes, no, and don't know.

### 2.3. Depressive Symptoms

The Center for Epidemiologic Studies Depression Scale (CES-D) was used to identify depressive symptoms [27]. The 20-item CES-D explores depressed mood, feelings of guilt and worthlessness, feelings of helplessness and hopelessness, psychomotor retardation, loss of appetite, and sleep disturbances. The answers to most questions were scored as 0 (rarely or never), 1 (sometimes), 2 (occasionally or more often), or 3 (most or all of the time). The final score (which had a maximum value of 60) was obtained by the sum of individual question scores, except for inverting the scores of 4 out of the 20 items.

### 2.4. Data Analysis

Data analysis was carried out using the computer software Statistical Package for Social Sciences (SPSS version 24.0; IBM Corp, Armonk, NY, USA). Descriptive statistics are presented as frequencies and percentages for categorical variables and means and standard deviations for quantitative variables. Comparisons included chi-square tests for categorical demographics and Mann–Whitney *U* tests for continuous variables. The Mann–Whitney *U* tests were chosen in lieu of independent sample *t*-tests because of the significant skewness in the variable distributions. A two-tailed probability value *p* < 0.05 was considered statistically significant.

## 3. Results

In total, 199 respondents were surveyed. The study sample included 67 men and 130 women. Age cohorts had an even distribution, wherein 35.9% were between 25 and 34 years of age, 33.8% were 35 and 44 years old, and 30.3% were 45 years and older. Half of the sample population resided in the south and southeast of the capital (*n* = 88, 45.6%); 52 participants lived in the state capital (26.9%); and 53 (27.5) lived in the north of the capital. Most of the participants (*n* = 126, 64.6%) had received college education or higher, and 35.4% (*n* = 69) had received less than college education. The groups were homogeneous and did not show any significant differences.

**Figure 1:**
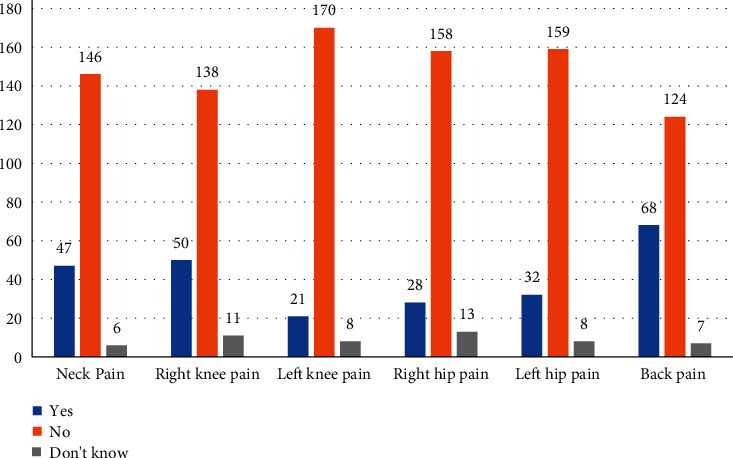
Prevalence of body pain at various sites.

Figure 1 outlines the counts for all possible answers to questions inquiring about bodily pain. The highest prevalence of pain was in the back, which accounted for 34.2% (*n* = 68 counts of yes answers) of the total, and 25.1% for the right knee pain (*n* = 50 counts of yes answers). The lowest “yes” responses were received for pain in the left knee (10.5%, *n* = 21).

Comparisons were drawn between those who scored <2 on the TMD-pain screener and those who scored ≥2. The results are presented in Table 1. Two significant differences were detected for those who reported TMD-related pain scores ≥2. These respondents reported significantly higher depression levels as measured by the CES-D depression scale (standardized Mann–Whitney *U* = 2.98, *p*=0.003) and significantly higher bodily pain scores (standardized Mann–Whitney *U* = 2.44, *p*=0.014).

### 3.1. Prevalence of TMD

According to the scoring protocol described in the Materials and Methods section [12], a score of two or higher in the short TMD-pain screener indicated painful TMD. As shown in Table 1, 26.8% of the sample (*n* = 42) reported painful TMD, with no statistically significant difference between men and women.

### 3.2. Predictors of TMD

As the TMD-pain screener and bodily pain scale measured similar constructs, a Spearman correlation was performed to assess the degree of overlap between the two variables. A small but significant correlation was observed (*r*_s_ = 0.22, *p*=0.002). A logistic regression analysis was performed to determine whether the level of bodily pain was an independent predictor of TMD-related pain, in addition to the CES-D. The logistic regression results shown in Table 2 indicate that bodily pain score did not add significantly to the prediction of TMD-related pain levels above and beyond the prediction using CES-D alone.

## 4. Discussion

The aim of this study was to estimate the prevalence of TMD in a clinical sample population of patients at the dental school in Kuwait and to investigate possible risk factors associated with cases identified as positive for TMD-related pain. TMD encompasses a broad spectrum of clinical symptoms and is considered to be the most common cause of nondental chronic orofacial pain. Joint noise and pain, as well as masticatory muscular pain, are among the most prominent symptoms of TMD [29].

The recruited sample population in this study tested the hypothesis that the prevalence of TMD-related pain among women is higher than that in men. We found that men and women did not report statistically significant differences in TMD-related (men 41.5%, women 58.5%, *p*=0.258); however, more women described symptoms of TMD-related pain. In population studies, female-to-male dominance among patients has been reported to approach a ratio of 10:1. Although a direct cause-and-effect relationship has not been established in the literature, some studies support the potential role of hormonal changes and female hormone receptors in this marked predilection [9, 18, 30]. The TMJ is a joint of special nature in the human body. Unlike the rest of the synovial joints, its articular surface is covered with fibrocartilage rather than hyaline cartilage, which superiorly withstands the shear forces created by occlusal load. Fibrocartilage is also known to have better healing ability and less age-related damage. However, fibrocartilage responds differently to sex hormones than hyaline cartilage, which might explain the elevated female predisposition to TMD [30]. It has been reported that painful TMD is more prevalent in women in their reproductive years [30, 31]. Animal studies investigating the expression of estrogen-binding sites within the joints demonstrated that 17-*β*-estradiol increases pain in inflamed joints through upregulation of hippocampal TRPV1 and that estrogen impacts nociception through the trigeminal ganglion [32, 33]. Others have proposed that women have increased sensitivity to biological stimuli [34]. Behaviorally, men and women are taught differently during their upbringing. While it might be acceptable for women to express pain and discomfort, men are more stoic [28].

Very few studies have reported the epidemiology of signs, symptoms, diagnoses, and associated demographic variables of TMD within the Middle East and the Arabian Peninsula. The findings of these studies are varied and inconsistent. In a report on prevalence and associations of TMD in Jordan using DC/TMD, Alrashdan et al. found that 16.3% out of 368 clinic sample participants aged 18–78 report having TMD-related pain. In another cross-sectional report on TMD signs and symptoms, Zwiri and Al-Omiri estimated the prevalence of at least one TMD symptom among a sample of 489 university students aged between 18 and 25 is approximately 50% [35, 36]. Nadershah screened 500 adult patients in Saudi Arabian primary care dental clinics by using a TMD-pain screener [37]. His analysis utilized a long screener, with a score of 3 or above to indicate a positive case of TMD-related pain. His findings revealed a 35% prevalence of TMD-related pain, with a predilection for females (42%) over males (28%). Although the proportions are similar, our findings, along with those of reported studies, demonstrate the variety of results available in the scientific literature and the inconsistencies in reporting these conditions.

Current evidence of TMD supports the psychosocial rather than the dental occlusion model for the etiology of TMD [23]. Psychosocial factors are among the recognized predisposing factors that play a role in the manifestation and extension of symptoms [1, 9, 11, 15, 38, 39]. Stress, anxiety, and depression are possible contributing factors that can lead to persistent muscular tension and hyperactivity, central dysregulation resulting in parafunctional habits, and potential occlusal disharmony [9, 13, 40]. We observed that participants who scored positive for TMD-related pain were more likely to have higher CES-D (depression) scores (OR = 1.01, 95% CI 1.02 to 1.10). This finding is consistent with the available evidence in the literature associating depression with the somatization of pain. People experiencing stress, depression, and anxiety more often exhibit symptoms of TMD pain [7, 9, 41].

Studies have demonstrated the coexistence of widespread body and joint pain in individuals with TMD [6, 12, 42–44]. Our data revealed that participants who scored positive for TMD complained of pain in distant joints. Although pain at distant sites did not predict TMD-related pain, we were able to demonstrate that the prevalence of pain in the back, right knee, and neck was high. Plesh et al. analyzed 2000–2005 National Health Interview Survey (NHIS) data of 189,992 participants [45]. The data examined the presence or absence of facial pain over the past 30-day period and the presence of pain in distant sites, such as head, neck, and lower back. Findings from this study revealed that among the sample population, 15.4% had painful headaches and migraines, 14.9% had neck pain, and 28% had lower back pain. The study did not examine the correlation between reports of TMD-related and distant pain. Another report examining 2009 NHIS pain data for adults, 18 years of age or above, revealed that among the 5% (*n* = 11.5 million) US adults who reported jaw or face pain within the preceding 3 months, associated neck pain, severe headache, migraine as well, and back pain persisted. In addition, 23.4% of individuals who reported jaw or face pain within the preceding 3 months also indicated that they had headache, neck, or nonaxial joint pain [46, 47]. The presence of dispersed joint pain signifies an explanatory model of central desensitization in describing the overlap of TMD with other pain conditions. It has been proposed that the interaction of environmental exposure with genetics results in a state of heightened hyperalgesia and susceptibility to negative effects, both of which are thought to contribute to the chronicity of pain in this unique patient population [46].

## 5. Conclusions

This study was conducted using a convenience sample at a dental school clinic. Our findings should be interpreted with the caveat that cases positive for TMD-related pain were not validated by clinical examination. Within the limitations of this study, it can be concluded that the prevalence of TMD-related pain is modest, evidence between associated variables and those who reported symptoms are consistent with the available literature.

Future locally conducted studies utilizing larger or population-based samples are warranted to help in developing a better understanding of the distribution of TMD-related symptoms and their associated variables within the region. Population demographics are unique, and local culture and practices certainly affect disease prevalence, risk, and outcomes. There is a need to conduct local population studies rather than encourage the universal application of the results of studies completed in distant geographies. This study can serve as a guide for future studies in the state of Kuwait and within the Gulf region, where additional research efforts and data can be pooled for a larger sample. Furthermore, clinical examination and radiological diagnostics will provide a more objective measure rather than the patients' recall of self-reported pain.

## Figures and Tables

**Table 1:** Descriptive statistics for the study variables.

		TMD <2 (*N* = 157), *n* (%)	TMD ≥2 (*N* = 42), *n* (%)	Total (*N* = 199), *n* (%)	Sig.
Sex					0.258
	Male	50 (32.1)	17 (41.5)	67 (28)	
	Female	106 (67.9)	24 (58.5)	130 (66)	

Governorate					
	State capital	41 (27.2)	11 (26.2)	52 (26.9)	0.982
	South and southeast capital	69 (45.7)	19 (45.2)	88 (45.6)	
	North capital	41 (27.2)	12 (28.6)	53 (27.5)	

Education					0.960
	Less than college	54 (35.3)	15 (35.7)	69 (35.4)	
	College or higher	99 (64.7)	27 (64.3)	126 (64.6)	

Age (years)					0.713
	25–34	58 (36.9)	13 (31.7)	71 (35.9)	
	35–44	51 (32.5)	16 (39.0)	67 (33.8)	
	45+	48 (30.6)	12 (29.3)	60 (30.3)	

TMD total score, M (SD)				0.74 (1.19)	
CES-D total score, M (SD)		12.69 (8.39)	17.55 (9.80)	13.71 (8.91)	0.003
Bodily pain total score, M (SD)		1.09 (1.41)	1.67 (1.59)	1.21 (1.46)	0.014

**Table 2:** Logistic regression results for the TMD pain model (*N* = 199).

Variables	B	S.E.	Wald	df	Sig.	OR	95% CI for OR	
							Lower	Upper
Bodily pain total score	0.22	0.12	3.55	1	0.059	1.25	0.99	1.57
CES-D total score	0.06	0.02	7.87	1	0.005	1.06	1.02	1.10

*Note.* OR = odds ratio. Overall model *χ*^*2*^ [2] = 12.97, *p*=0.002.

## Data Availability

The raw data used to support the findings of this study are available from the corresponding author upon request.
